# Evaluating Lipid‐Lowering Drug Targets for Parkinson's Disease Prevention with Mendelian Randomization

**DOI:** 10.1002/ana.25880

**Published:** 2020-09-07

**Authors:** Dylan M. Williams, Sara Bandres‐Ciga, Karl Heilbron, David Hinds, Alastair J. Noyce, The 23andMe Research Team, The International Parkinson's Disease Genomics Consortium

**Affiliations:** ^1^ MRC Unit for Lifelong Health and Ageing at UCL University College London London UK; ^2^ Department of Medical Epidemiology & Biostatistics Karolinska Institutet Stockholm Sweden; ^3^ Molecular Genetics Section, Laboratory of Neurogenetics National Institute on Aging, National Institutes of Health Bethesda MD USA; ^4^ Instituto de Investigación Biosanitaria de Granada Granada Spain; ^5^ 23andMe, Inc Sunnyvale CA USA; ^6^ Preventive Neurology Unit, Wolfson Institute of Preventive Medicine Queen Mary University of London London UK; ^7^ Department of Clinical and Movement Neurosciences UCL Institute of Neurology London UK

## Abstract

Long‐term exposure to lipid‐lowering drugs might affect Parkinson's disease (PD) risk. We conducted Mendelian randomization analyses where genetic variants indexed expected effects of modulating lipid‐lowering drug targets on PD. Statin exposure was not predicted to increase PD risk, although results were not precise enough to support benefits for prevention clearly (odds ratio [OR] = 0.83; 95% confidence interval [CI] = 0.65, 1.07). Other target results were null, except for variants indicating Apolipoprotein‐A5 or Apolipoprotein‐C3 inhibition might confer protection. These findings suggest peripheral lipid variation may not have a prominent role in PD etiology, but some related drug targets could influence PD via alternate pathways. ANN NEUROL 2020;88:1043–1047

Epidemiological studies have found inconsistent associations between statin use and Parkinson's disease (PD) risk, indicating that exposure might provide neuroprotection^1^, ^2^, ^3^ or heighten PD risk.^4^, ^5^ However, such observational studies are affected by biases that limit causal inference, such as confounding and reverse causation. Assessing the potential of statins or other lipid‐lowering agents for PD prevention robustly in long‐duration randomized controlled trials would be challenging and, hence, the use of other study designs is warranted to examine whether exposure to lipid‐lowering drugs may mitigate or increase PD risk.

Genetic variation can be used to predict the effects of long‐term drug exposure on disease risk. Variation in the vicinity of a protein‐coding gene can affect protein production or function in a similar way to the therapeutic modulation of the same protein with drugs. Associations of so‐called *cis‐*acting variants with traits are not prone to conventional confounding or reverse causation due to Mendelian randomization principles (*cis*‐MR).^6^ In this study, we used *cis‐*MR to examine whether PD risk may be affected by long‐term exposure to several drug classes related to treatment of primary or familial hypercholesterolemia.

## Methods

### *Study Design*

We conducted two‐sample MR analyses.^7^ In *cis*‐MR models, we principally addressed 4 licensed lipid‐lowering drug classes that reduce circulating low‐density lipoprotein cholesterol (LDL‐C; Table 1). Given previous evidence that lower Apolipoprotein B (ApoB) concentrations may increase PD risk,^8^ and that ApoB is a major transporter of triglycerides as well as LDL‐C, we also addressed a selection of other drug targets that reduce circulating triglycerides and are targeted by licensed or novel therapeutics. For context, we conducted conventional MR models to estimate whether PD risk is affected by long‐term reductions in circulating LDL‐C, triglycerides, and ApoB (the lipoprotein involved in LDL‐C and triglyceride transport; ie, estimating expected consequences for PD risk from reductions in these traits irrespective of the means of reduction), and not necessarily due to any specific drug class.

**TABLE 1:** Information on Lipid‐Lowering Drug Targets under Investigation

Target Groups by Primary Pharmacological Action	Examples of Drugs/Class	Target	Gene Encoding Target^a^ ^,^ ^b^	Gene ID^a^
Reduced circulating LDL‐C	Statins	HMG‐coA reductase (HMGCR)	*HMGCR*	5006
	Evolocumab	Proprotein convertase subtilisin/kexin type 9 (PCSK9)	*PCSK9*	20001
	Mipomersen	Apo‐B 100 messenger RNA (ApoB‐100)	*APOB*	603
	Ezetimibe	Nieman Pick C1‐like protein 1 (NPC1L1)	*NPC1L1*	7897
Reduced VLDL‐C / triglycerides	Fibrates	Peroxisome proliferator‐activated receptor alpha (PPARα)	*PPARA*	9232
	Evinacumab (investigational)	Angiopoietin‐related protein 3 (ANGPTL3)	*ANGPTL3*	491
	Investigational target	Lipoprotein lipase (LPL)	*LPL*	6677
	Investigational target	Apolipoprotein A‐5 (ApoA5)^c^	*APOA5*	17288
	Investigational target	Apolipoprotein C‐3 (ApoC3)^c^	*APOC3*	610

^a^ Gene symbols and IDs from the HUGO Gene Nomenclature Committee.

^b^ Selection of variants within/near genes used start / stop coordinates of gene positions based on Human Genome reference release GRCh37.

^c^ Due to the proximity of the genes encoding ApoA5 and ApoC3, and the likelihood of high low density between variants in the region, variants in the vicinity of these genes were combined in MR models.

LDL‐C = low‐density lipoprotein cholesterol; MR = Mendelian randomization; VLDL‐C = very low‐density lipoprotein cholesterol.

### *SNP‐Lipid Associations*

To validate that genetic variants index physiological responses expected from the use of corresponding drugs, we combined genetic association statistics from specific gene regions of interest from 3 large genome‐wide association studies (GWAS) of LDL‐C, triglycerides, and ApoB (N per SNP‐lipid association = 14,004 to 295,826; see Supplementary Table S1).^9^, ^10^, ^11^

### *SNP‐PD Associations*

SNP‐lipid association statistics were harmonized with corresponding SNP‐PD risk estimates from several large PD case‐control GWAS samples using data from 23andMe and the International Parkinson's Disease Genomics Consortium (total N = 37,688 cases and 981,372 controls; see Supplementary Table S2).^12^ PD ascertainment was via self‐report in the 23andMe sample and via clinical assessment in all other samples. Lipid and PD GWAS sample overlap is noted under Supplementary Table S2.

### *Statistics*

We conducted 2 types of *cis*‐MR models, primarily using adjusted estimates from correlated variants within gene regions,^13^ and with secondary analyses using only uncorrelated variants to check consistency. Effects of general, long‐term reductions in LDL‐C, triglycerides, and ApoB on PD risk were estimated with conventional 2‐sample MR methods: (i) the inverse variance weighted (IVW) approach primarily, and (ii) weighted median and MR Egger methods (see Supplementary Table S3 for details).^14^, ^15^ All results were expressed as PD risk per standard deviation (SD) lower LDL‐C, triglycerides, or ApoB, so that findings are indicative of the use of corresponding lipid‐lowering therapeutics.

Power calculations are described in Supplementary Table S4. Analyses were conducted in R with packages “*TwoSampleMR*” and “*MendelianRandomization*.”^16^, ^17^

### *Ethics*

This study used existing summary GWAS data, so separate ethical approval was not required (all prior studies had ethical approval in accordance with the Declaration of Helsinki).

## Results

Descriptive statistics and association data for SNPs used in the analyses are provided in Supplementary Tables S5 and S6. In MR models for LDL‐lowering drug targets (Table 2), the point estimate for HMG‐CoA reductase (HMGCR) inhibition (indicative of statin use) was consistent with modest neuroprotection, but the confidence interval (CI) was wide and included the null. Results for PCSK9 and NPC1L1 inhibition were close to the null. The estimate for ApoB silencing (via mipomersen exposure, for example) was on the side of harm but its CI also included the null. These findings were similar, but less precise, when repeated with secondary *cis‐*MR models.

**TABLE 2:** *cis‐*MR Model Estimates for the Effects of Modulating Lipid‐Lowering Drug Targets on PD Risk

	Gene / target	Primary Models^a^				Secondary Models^b^		
		N SNPs^a^	N PCs^a^	OR	95% CI	N SNPs	OR	95% CI
Estimates for LDL‐lowering targets weighted by LDL‐C	*APOB*	83	4	1.16	(0.97, 1.39)	3	1.18	(0.97, 1.42)
	*HMGCR*	64	3	0.83	(0.65, 1.07)	1	0.92	(0.66, 1.28)
	*NPC1L1*	91	7	0.92	(0.65, 1.30)	1	0.91	(0.54, 1.51)
	*PCSK9*	150	7	1.06	(0.90, 1.25)	2	1.15	(0.84, 1.57)
Estimates for VLDL‐lowering targets weighted by triglycerides	*ANGPTL3*	43	3	0.88	(0.66, 1.17)	1	0.93	(0.69, 1.24)
	*APOA5/APOC3*	132	4	0.82	(0.72, 0.94)	2	0.89	(0.79, 1.00)
	*APOB*	83	3	1.00	(0.65, 1.53)	1	0.94	(0.68, 1.29)
	*LPL*	164	6	1.02	(0.87, 1.20)	1	0.89	(0.74, 1.07)
	*PPARA*	185	14	0.92	(0.64, 1.33)	N/A^c^		
Estimates for all targets weighted by ApoB	*ANGPTL3*	43	6	1.24	(0.88, 1.76)	1	1.19	(0.71, 2.01)
	*APOA5/APOC3*	140	3	0.76	(0.63, 0.91)	1	0.79	(0.67, 0.93)
	*APOB*	85	4	1.10	(0.89, 1.36)	1	1.09	(0.90, 1.31)
	*HMGCR*	64	3	0.86	(0.68, 1.09)	1	0.87	(0.65, 1.16)
	*LPL*	170	6	0.98	(0.79, 1.22)	1	0.93	(0.69, 1.26)
	*NPC1L1*	89	6	0.93	(0.69, 1.27)	N/A^c^		
	*PCSK9*	162	7	1.11	(0.94, 1.32)	2	1.06	(0.90, 1.25)
	*PPARA*	183	13	0.96	(0.72, 1.27)	N/A^c^		

^a^ In primary models using the inverse variance weighted method with PC analysis, the numbers of SNPs indicated were used to derive PCs, which are the basis of instruments for the MR estimate. MR models included a subset of these PCs, which were expected to explain 90% of the variation in lipid concentrations attributable to variants at these loci when combined (κ = 0.9). Primary and secondary methods are described further and compared in Supplementary Table S3.

^b^ Where secondary MR models included a single variant, MR estimates were based on single Wald estimators. Where two or more variants were included, estimates were based on the standard inverse variance weighted method approach.

^c^ Estimates for secondary models were not derived for drug targets in instances when no SNP in the region had a *p* value for association with the relevant circulating lipid under 5 × 10^−8^.

ApoB = apolipoprotein B; CI = confidence interval; *cis*‐MR = conventional confounding or reverse causation due to Mendelian randomization principles; LDL‐C = low‐density lipoprotein cholesterol; MR = Mendelian randomization; OR = odds ratio; PC = principal component; PD = Parkinson's disease; SNP = single nucleotide polymorphism; VLDL = very low‐density lipoprotein cholesterol.

For triglyceride‐reducing drug targets (Table 2), findings were centred on, or close to, the null for *APOB* (ApoB silencing indexed here via a reduction in triglycerides, rather than LDL‐C), *PPARA* (indicative of fibrate use), *ANGPTL3* (indicative of angiopoietin‐like 3 inhibitors, an investigational class), and *LPL* (indicative of investigational lipoprotein lipase activators). However, many of these results were estimated imprecisely. In contrast, ApoA5 / ApoC3 modulation was predicted to lower PD risk, and estimated with higher precision (odds ratio (OR) per SD lower triglycerides from ApoA5 / ApoC3 modulation = 0.82; 95% CI = 0.72, 0.94; *p* = 0.005).

**FIGURE 1 1:**
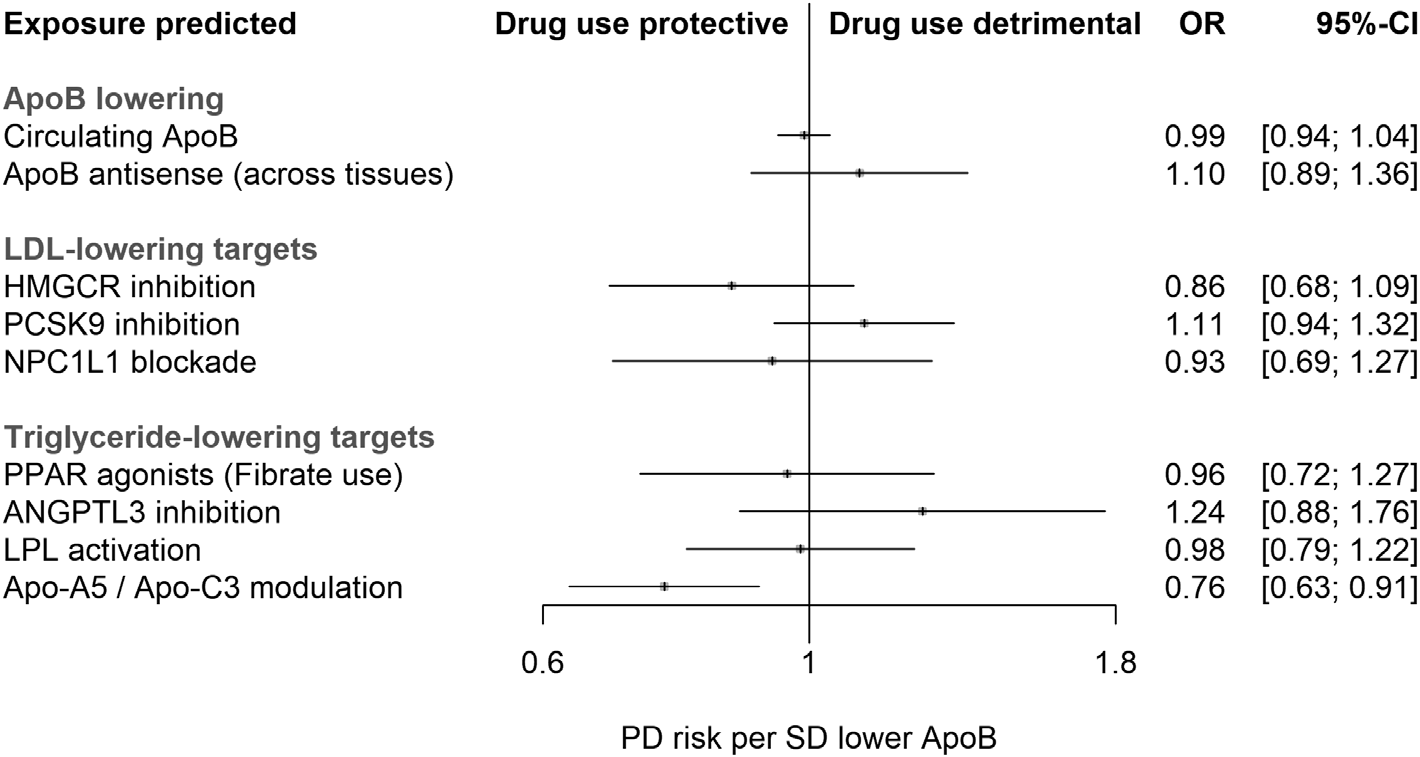
MR estimates for the effects of modulating lipid‐lowering drug targets on PD risk, weighted by circulating ApoB. ApoB = apolipoprotein B; CI = confidence interval; LDL = low‐density lipoprotein; MR = Mendelian randomization; OR = odds ratio; PD = Parkinson's disease; SD = standard deviation.

Both sets of drug target models (for LDL‐lowering and triglyceride‐lowering targets) were repeated with genetic indexing of the degree to which all targets under investigation lower ApoB in circulation, reflecting combined reductions in LDL‐C and very low‐density lipoprotein cholesterol, a major transporter of triglycerides (Table 2, Fig. 1). Results for all other LDL‐lowering and triglyceride‐lowering targets were again close to the null, with the exception of ApoA5 / ApoC3 modulation. The smallest *p* value for genotype‐PD associations in the region was 5.9 × 10^−5^ for rs4520 in *APOC3*.

In standard MR models that quantified the impact of lowering LDL‐C, triglycerides, or ApoB on PD risk, there was no notable evidence supporting effects (Supplementary Table S7). Results from main and alternate methods were consistently null for LDL‐C. Point estimates for an effect of reducing circulating triglycerides were narrowly on the side of harm but close to the null in all models. Estimates for circulating ApoB was not estimated to affect PD risk by any MR method, and were not substantially different from estimates of an effect of specific inhibition of ApoB function (averaged across any tissue‐specific effects) on PD from drug target models of *APOB* (see Fig. 1).

Most MR models were expected to have ≥ 80% power to detect ORs of 0.88 or lower per SD lipid reduction (see Supplementary Table S4).

## Discussion

This is the largest MR study to have investigated lipid lowering and the modulation of corresponding drug targets in relation to PD risk. Null findings for circulating concentrations of LDL‐C, triglycerides, and ApoB refute a role of peripheral lipid modulation in the etiology of PD, providing a timely context to conflicting evidence from case‐control or prospective studies of lipid biomarkers or statin use.^1^, ^2^, ^3^, ^4^, ^8^ Two previous studies have examined similar questions with MR.^8^, ^18^ Benn et al did not find effects of lowering LDL‐C and modulating HMGCR and PCSK9 on PD risk, but their analyses lacked power to detect effects given MR models based on ≤ 579 PD cases.^18^ Fang et al reported very modest associations of higher genetically indexed LDL‐C (OR = 0.96; 95% CI = 0.92, 0.99) and triglycerides (OR = 0.94; 95% CI = 0.89, 1.00) with lower PD risk in a similar sample used for this analysis.^8^ However, these results may have been false positives for technical reasons (too many variants were included in MR models because some are correlated).

Results for specific targets could still indicate benefits (or risks) for PD of using related therapeutics, as their modulation may have physiological effects beyond lipid lowering. Hence, our result for *HMGCR*, on the side of neuroprotection, does not rule out possible benefits of on‐target effects of using statins for PD prevention via alternative mechanisms. Concomitantly, this evidence opposes findings from large observational studies of health insurance records that have suggested statin exposure may increase PD risk.^4^, ^5^ We also identified strong genetic evidence implicating one or more genes in the apolipoprotein gene cluster on chromosome 11 in PD etiology. However, closely situated variants could have *cis‐*acting effects on multiple proteins in the region (horizontal pleiotropy) in addition to ApoA5 and ApoC3, and / or be correlated with variants affecting other proteins via linkage. Thus, the current analyses do not pinpoint the exact protein(s) of relevance for PD among several possible candidates encoded from genes in this region. We also note that larger genetic association studies will be required to confirm that this finding did not result from chance.

The major strengths of this study are the use of a very large sample of genetic data and an MR design for causal inference. Key limitations include: (1) estimation of on‐target effects of drug use only (these models do not estimate potential off‐target effects); (2) lack of precision to robustly identify modest but potentially meaningful effects for some targets, including HMGCR inhibition; and (3) the possibility of survival bias, given that dyslipidaemias increase mortality prior to late‐onset PD, which might bias findings toward spurious inverse associations with PD risk. We also note that our findings relate primarily to PD incidence, and not to the potential for these drug targets to mitigate PD progression among cases.

Larger scale MR studies, and other forms of pharmacoepidemiology, would help to further evaluate the role of statin exposure in PD risk and progression and assess the potential for neuroprotection from ApoA5 and / or ApoC3 modulation. Using tissue‐specific expression quantitative trait loci in future *cis‐*MR analyses may help to resolve questions regarding apolipoproteins coded by the *APOA5* / *APOC3* cluster in relation to the pathogenesis of PD.

## Author Contributions

D.M.W., S.B.C., and A.J.N. conceived of the study. All authors contributed to its design. D.M.W., K.H., D.H., the IPDGC, and the 23andMe Research Team contributed to data analyses. All authors contributed to the drafting of the article's text and approved the manuscript for publication. Members of the IPDGC and the 23andMe Research Team are listed in Supplementary Table S8.

## Potential Conflicts of Interest

The authors declared no conflict of interest.

## Supporting information


**Appendix S1.** Supporting information.

## References

[1] Undela K , Gudala K , Malla S , Bansal D . Statin use and risk of Parkinson's disease: a meta‐analysis of observational studies. J Neurol 2013;260:158–165. 22820685 10.1007/s00415-012-6606-3

[2] Sheng Z , Jia X , Kang M . Statin use and risk of Parkinson's disease: a meta‐analysis. Behav Brain Res 2016;309:29–34. 27131781 10.1016/j.bbr.2016.04.046

[3] Yan J , Qiao L , Tian J , et al. Effect of statins on Parkinson's disease: a systematic review and meta‐analysis. Medicine 2019;98:e14852. 30896628 10.1097/MD.0000000000014852 PMC6709163

[4] Jeong S‐M , Jang W , Shin DW . Association of statin use with Parkinson's disease: dose–response relationship. Mov Disord 2019;34:1014–1021. 30938893 10.1002/mds.27681

[5] Liu G , Sterling NW , Kong L , et al. Statins may facilitate Parkinson's disease: insight gained from a large, national claims database. Mov Disord 2017;32:913–917. 28370314 10.1002/mds.27006 PMC5466869

[6] Swerdlow DI , Kuchenbaecker KB , Shah S , et al. Selecting instruments for Mendelian randomization in the wake of genome‐wide association studies. Int J Epidemiol 2016;45:1600–1616. 27342221 10.1093/ije/dyw088 PMC5100611

[7] Burgess S , Butterworth A , Thompson SG . Mendelian randomization analysis with multiple genetic variants using summarized data. Genet Epidemiol 2013;37:658–665. 24114802 10.1002/gepi.21758 PMC4377079

[8] Fang F , Zhan Y , Hammar N , et al. Lipids, Apolipoproteins, and the risk of Parkinson disease: a prospective cohort study and a Mendelian randomization analysis. Circ Res 2019;125:643–652. 31382822 10.1161/CIRCRESAHA.119.314929

[9] Willer CJ , Schmidt EM , Sengupta S , et al. Discovery and refinement of loci associated with lipid levels. Nat Genet 2013;45:1274–1283. 24097068 10.1038/ng.2797 PMC3838666

[10] Kettunen J , Demirkan A , Würtz P , et al. Genome‐wide study for circulating metabolites identifies 62 loci and reveals novel systemic effects of LPA. Nat Commun 2016;7:11122. 27005778 10.1038/ncomms11122 PMC4814583

[11] Liu DJ , Peloso GM , Yu H , et al. Exome‐wide association study of plasma lipids in >300,000 individuals. Nat Genet 2017;49:1758–1766. 29083408 10.1038/ng.3977 PMC5709146

[12] Nalls MA , Blauwendraat C , Vallerga CL , et al. Identification of novel risk loci, causal insights, and heritable risk for Parkinson's disease: a meta‐analysis of genome‐wide association studies. Lancet Neurol 2019;18:1091–1102. 31701892 10.1016/S1474-4422(19)30320-5 PMC8422160

[13] Burgess S , Zuber V , Valdes‐Marquez E , et al. Mendelian randomization with fine‐mapped genetic data: choosing from large numbers of correlated instrumental variables. Genet Epidemiol 2017;41:714–725. 28944551 10.1002/gepi.22077 PMC5725678

[14] Bowden J , Davey Smith G , Burgess S . Mendelian randomization with invalid instruments: effect estimation and bias detection through egger regression. Int J Epidemiol 2015;44:512–525. 26050253 10.1093/ije/dyv080 PMC4469799

[15] Bowden J , Davey Smith G , Haycock PC , Burgess S . Consistent estimation in Mendelian randomization with some invalid instruments using a weighted median estimator. Genet Epidemiol 2016;40:304–314. 27061298 10.1002/gepi.21965 PMC4849733

[16] Hemani G , Zheng J , Elsworth B , et al. The MR‐base platform supports systematic causal inference across the human phenome. Elife 2018;7:e34408. 29846171 10.7554/eLife.34408 PMC5976434

[17] Yavorska OO , Burgess S . MendelianRandomization: an R package for performing Mendelian randomization analyses using summarized data. Int J Epidemiol 2017;46:1734–1739. 28398548 10.1093/ije/dyx034 PMC5510723

[18] Benn M , Nordestgaard BG , Frikke‐Schmidt R , Tybjærg‐Hansen A . Low LDL cholesterol, PCSK9 and HMGCR genetic variation, and risk of Alzheimer's disease and Parkinson's disease: Mendelian randomisation study. BMJ 2017;357:j1648. 28438747 10.1136/bmj.j1648 PMC5421439

