# Supplementary material for: Evaluating Lipid‐Lowering Drug Targets for Parkinson's Disease Prevention with Mendelian Randomization
Source: Ann Neurol. 2020 Sep 7;88(5):1043–7. doi: 10.1002/ana.25880 (PMC7693098; doi:10.1002/ana.25880)
Supplement: Supplementary file 1 — Appendix S1. Supporting information. [file ANA-88-1043-s001.docx]

**Evaluating lipid-lowering drug targets for Parkinson’s disease prevention with Mendelian randomization**

Dylan M. Williams PhD_,_ Sara Bandres-Ciga PhD, Karl Heilbron PhD, the 23andMe Research Team, the International Parkinson’s Disease Genomics Consortium, David Hinds PhD^,^ Alastair J Noyce MRCP, PhD

***Supplemental tables***

**Table of Contents**

[Supplemental table 1: Information on studies used to instrument variation in lipid concentrations 2](#_Toc45542250)

[Supplemental table 2 – Summary details on PD case-control samples meta-analysed for this MR study 3](#_Toc45542251)

[Supplemental table 3: Summary details of the different applications of MR methods used in analyses 5](#_Toc45542252)

[Supplemental table 4 – Power calculations to illustrate expected detectable odds ratios (in either direction), given the strength of instruments for the traits and the sample size in MR models 6](#_Toc45542253)

[Supplemental table 5: information on sentinel gene-specific variants in regions used to assess the effects of drug targets on PD in *cis-*MR models 7](#_Toc45542254)

[Supplemental table 6: information on variants used to assess the effects of lowering circulating LDL-C, triglycerides and ApoB on PD with standard MR models 9](#_Toc45542255)

[Supplemental table 7 – MR estimates for the effects of lowering LDL-C, triglycerides and ApoB on PD risk 15](#_Toc45542256)

[Supplemental table 8: group authorship members 16](#_Toc45542257)

# **Supplemental table 1:** Information on studies used to instrument variation in lipid concentrations

| **Consortium** | **Reference DOI** | **Biomarkers studied** | **Design** | **# SNPs in GWAS** | **Sample size** | **Lipid assaying** |
| --- | --- | --- | --- | --- | --- | --- |
| GLGC | 10.1038/ng.2797 | LDL-C and triglycerides | GWAS | ~ 2.4 million | 173041 | Direct assay or Friedewald estimation |
| GLGC | 10.1038/ng.3977 | LDL-C and triglycerides | Exome-wide association study | ~ 242000 | 295826 | Direct assay or Friedewald estimation |
| Magnetic | 10.1038/ncomms11122 | LDL-C, triglycerides, ApoB | GWAS | ~ 11.8 million | 21559 | Nuclear magnetic resonance spectroscopy |

*Abbreviations: GLGC ­­– global lipid genomics consortium*

Footnotes:

1. To maximise the coverage and precision of SNP-lipid estimates used in MR models, we appended SNP-lipid estimates together from the three sources described in the table (these samples partly overlapped and could not be meta-analysed). Where the same SNP-lipid association was present in more than one sample, we retained the estimate from the largest sample. All participants were of European ancestry.

# **Supplemental table 2** – Summary details on PD case-control samples meta-analysed for this MR study

| **Study** | **Cases (N)** | **Controls (N)** | **Female cases (%)** | **Female controls (%)** | **Case age onset (mean (SD); years)** | **Control age, last exam (mean (SD); years)** | **Case ascertain.** | **Control**  **ascertain.** |
| --- | --- | --- | --- | --- | --- | --- | --- | --- |
| 23andMe PDWBS sample | 6,476 | 302,042 | Sample details in Chang *et al,* Nat Genet 2017;49(10):1511 | | | | Self-report | Self-report |
| 23andMe post-PDWBS sample | 2,448 | 571,411 | 39.1 | 54.9 | 61.8 (10.9) | 51.5 (16.8) | Self-report | Self-report |
| Baylor College / Uni. Maryland | 769 | 195 | 33.8 | 69.7 | 64.8 (10.1) | 65.5 (8.3) | Clinic | Clinic & self-report |
| Finnish Parkinson's | 386 | 493 | 45.9 | 78.9 | 55.3 (5.6) | 92.4 (3.9) | Clinic | Population-based |
| Harvard Biomarker Study | 527 | 472 | 34.4 | 61.7 | 66.3 (10.1) | 69.9 (9.0) | Clinic | Clinic & self-report |
| IPDGC (Nalls et al. 2014 discovery sample) | 13,708 | 95,282 | Study-level data in Nalls *et al*. Nat Genet 2014;46(9):989-993 | | | | Clinic or self-report | Clinic or self-report |
| McGill Parkinson's | 582 | 905 | 34.5 | 48.4 | 65.7 (9.8) | 55.8 (10.7) | Clinic | Clinic & self-report |
| Oslo PD Study | 476 | 462 | 35.7 | 42.2 | 65.3 (9.3) | 61.9 (11.1) | Clinic | Clinic & self-report |
| NeuroX study | 5,851 | 5,866 | Study-level data in Nalls *et al*. Nat Genet 2014;46(9):989-993 | | | | Clinic | Clinic & self-report |
| PD Biomarker's Program | 512 | 282 | 38.7 | 51.1 | 64.5 (9.4) | 62.2 (10.7) | Clinic | Clinic & self-report |
| Parkinson's Progression Markers Initiative | 363 | 165 | 33.1 | 33.3 | 64.2 (9.7) | 63.8 (10.6) | Clinic | Clinic & self-report |
| System Genomics of PD | 1,169 | 968 | 35.2 | 53.9 | 59.9 (10.9) | 66.6 (9.7) | Clinic | Clinic & self-report |
| Spanish Parkinson's | 2,110 | 1,333 | 43.1 | 54.4 | 63.9 (12.5) | 64.0 (12.6) | Clinic | Clinic & self-report |
| Tubingen Parkinson's Disease cohort | 666 | 542 | 36.0 | 57.9 | 59.9 (11.3) | 67.5 (8.4) | Clinic | Clinic & self-report |
| UK PDMED | 1,025 | 655 | 32.8 | 72.7 | NA | NA | Clinic | Clinic & self-report |
| Vance | 620 | 299 | 27.7 | 50.8 | 77.5 (8.4) | 82.0 (12.8) | Clinic | Clinic & self-report |
| Total in meta-analysis | 37,688 | 981,372 |  |  |  |  |  |  |

Footnotes:

1. Full ascertainment details are shown in table S1 of Nalls *et al*, 2019. *Lancet Neurol* 2019; 18: 1091–102. All participants were of European ancestry. The sole difference with the sample used in this study is that we only used data from case-control samples in the current MR analyses, thereby excluding data on genetic associations with proxy risk of PD from the UK Biobank study, which were also included in the previous IPDGC GWAS by Nalls *et al*.
2. In the presence of weak instruments, overlap between samples used to estimate SNP-exposure and SNP-outcome associations in two-sample MR may bias results towards the confounded observational exposure-outcome association (see Burgess *et al.* Genet Epidemiol 2016;40(7):597-608). However, overlap between participants of the GWAS of lipids and PD risk is likely to be nominal because the majority of lipid GWAS participants were in cohort studies or case-control studies related to coronary heart disease genetics, whereas PD GWAS participants were predominantly from bespoke case-control studies of PD genetics or derived from the 23andMe user base (which is not included in any of the three lipid GWAS).

# Supplemental table 3: Summary details of the different applications of MR methods used in analyses

| **Application in MR analyses** | **Variant selection range** | **LD clumping strategy** | **Handling of potentially correlated MR estimates** | **Assessment of violation by horizontal pleiotropy** |
| --- | --- | --- | --- | --- |
| Primary drug target analyses (*cis*-MR) | Within gene start/stop coordinates ± 100 kilobases | SNPs in entire selection region clumped, retaining SNPs with pairwise r2<0.6 (this retains some SNPs with low to moderate correlations) | Use of principal component analysis on a weighted matrix of correlations between SNPs, which accounts for residual correlations between the estimators subsequently used in MR modelling (IVW using principal component analysis; see doi:10.1002/gepi.22077). Variant correlations were estimated using reference data from individuals of European ancestry in the 1000 Genomes project, phase three (see doi:10.1038/nature15393) | Not assessed directly in MR modelling, but horizontal pleiotropy is minimised by using variants solely within the vicinity of the genes encoding drug targets of interest |
| Secondary drug target analyses (*cis*-MR) | Within gene start/stop coordinates ± 100 kilobases | SNPs in entire selection region clumped, retaining SNPs with lipid association *P* values <5×10^-8^ and pairwise *r^2^*<0.001 | No correlated estimates are retained for MR models | As for primary drug target analyses |
| Instrumenting effects of modulating circulating lipid concentrations on PD -- not specific to any single drug target (standard MR) | Genome-wide | SNPs with lipid association *P* values <5×10^-8^  clumped in 10,000kb windows, retaining SNPs with pairwise *r^2^*<0.001 | No correlated estimates are retained for MR models | Not assessed or adjusted for in the primary MR method applied here (IVW with first order weights). Alternative MR methods – weighted median and MR Egger approaches – estimate effects while accounting for some degree of horizontal pleiotropy. The MR Egger intercept test indicates whether there is averaged (unbalanced) effects of bias from horizontal pleiotropy. |

# **Supplemental table 4** – Power calculations to illustrate expected detectable odds ratios (in either direction), given the strength of instruments for the traits and the sample size in MR models

|  |  | **Power (%) to detect the following odds ratios per SD difference in the trait:** | | | | | |
| --- | --- | --- | --- | --- | --- | --- | --- |
| **Trait** | **% variance in trait explained by instruments (R^2^)** | **0.85** | **0.90** | **0.95** | **1.05** | **1.10** | **1.15** |
| LDL-C | 14.6 | 100 | 100 | 96.2 | 94.5 | 100 | 100 |
| Trigs | 11.7 | 100 | 100 | 91.7 | 88.9 | 100 | 100 |
| ApoB | 8.6 | 100 | 100 | 81.8 | 77.9 | 100 | 100 |
| Single region analyses, e.g. *PCSK9* weighted by effect on LDL-C * | 1.4 | 95.6 | 66.2 | 21.1 | 19.5 | 57.5 | 88.4 |

Based on a sample of 37,688 cases with 1:26 case-control ratio and α = 0.05

Footnotes:

1. Variance in LDL-C explained by a single, sentinel SNP in the *PCSK9* gene region reported in one of the previous GWAS of lipids.^1^ The use of this variance in calculations is likely to slightly underestimate power in the current analysis, which is based on the use of more genetic variation in the gene region (and hence models higher variance in LDL-C attributable to PCSK9 modulation).
2. Power was high for the analyses of circulating LDL-C, triglycerides and ApoB in relation to PD because of desirable instrument strength provided by SNPs indexing these traits in models, as indicated by R^2^ values for top GWAS hits ranging from 8.6 to 14.6%. In contrast, single gene region analyses may have had insufficient power for the detection of small or moderate associations, which could still have meaningful magnitudes for PD risk. This is particularly the case where these regions determine less difference in lipid variation than *PCSK9* does for circulating LDL-C, including *HMGCR* and *NPC1L1*.

# Supplemental table 5: information on sentinel gene-specific variants in regions used to assess the effects of drug targets on PD in *cis-*MR models

| **SNP** | **Gene** | **Effect**  **allele** | **Other**  **allele** | **EAF** | **Beta**  **exposure** | **SE**  **exposure** | ***P* value**  **exposure** | **F**  **exposure** | **N**  **exposure** | **Beta**  **outcome** | **SE**  **outcome** | ***P* value**  **outcome** |
| --- | --- | --- | --- | --- | --- | --- | --- | --- | --- | --- | --- | --- |
|  |  |  |  |  |  |  |  |  |  |  |  |  |
| *SNPs used in LDL-weighted drug target analyses* | | | |  |  |  |  |  |  |  |  |  |
| rs12714264 | *APOB* | A | T | 0.832 | 0.127 | 0.005 | 1.00E-200 | Infinite | 172866 | -0.032 | 0.014 | 0.024 |
| rs4341893 | *APOB* | A | G | 0.309 | 0.060 | 0.004 | 1.00E-200 | Infinite | 170089 | 0.004 | 0.011 | 0.731 |
| rs11206510 | *PCSK9* | C | T | 0.167 | -0.070 | 0.004 | 1.00E-200 | Infinite | 294565 | 0.021 | 0.012 | 0.086 |
| rs2495477 | *PCSK9* | T | C | 0.600 | 0.064 | 0.005 | 7.29E-30 | 129.0 | 80151 | 0.001 | 0.011 | 0.939 |
| rs10062361 | *HMGCR* | T | C | 0.235 | 0.069 | 0.004 | 1.00E-200 | Infinite | 173022 | 0.006 | 0.012 | 0.628 |
| rs2073547 | *NPC1L1* | G | A | 0.194 | 0.049 | 0.005 | 1.92E-21 | 90.4 | 169889 | 0.005 | 0.013 | 0.709 |
|  |  |  |  |  |  |  |  |  |  |  |  |  |
| *SNPs used in triglyceride-weighted drug target analyses* | | | |  |  |  |  |  |  |  |  |  |
| rs4587594 | *ANGPTL3* | G | A | 0.690 | 0.069 | 0.004 | 3.50E-82 | 369.1 | 177772 | 0.005 | 0.010 | 0.606 |
| rs676210 | *APOB* | G | A | 0.769 | 0.073 | 0.004 | 3.28E-71 | 318.7 | 177782 | 0.005 | 0.012 | 0.681 |
| rs12280753 | *APOA5/APOC3* | T | C | 0.067 | 0.193 | 0.006 | 1.22E-179 | Infinite | 177747 | 0.026 | 0.019 | 0.169 |
| rs7350481 | *APOA5/APOC3* | T | C | 0.098 | 0.225 | 0.007 | 1.00E-200 | Infinite | 177677 | 0.025 | 0.019 | 0.186 |
| rs12678919 | *LPL* | A | G | 0.879 | 0.170 | 0.006 | 1.82E-199 | Infinite | 177750 | 0.020 | 0.016 | 0.214 |
|  |  |  |  |  |  |  |  |  |  |  |  |  |
| *SNPs used in ApoB-weighted drug target analyses* | | | |  |  |  |  |  |  |  |  |  |
| rs1367117 | *APOB* | A | G | 0.288 | 0.109 | 0.011 | 9.99E-22 | 91.9 | 20686 | -0.009 | 0.010 | 0.395 |
| rs11591147 | *PCSK9* | T | G | 0.031 | -0.438 | 0.035 | 2.50E-34 | 150.0 | 15270 | 0.036 | 0.043 | 0.405 |
| rs2495477 | *PCSK9* | G | A | 0.419 | -0.062 | 0.011 | 3.14E-08 | 30.6 | 20687 | -0.001 | 0.011 | 0.939 |
| rs10056811 | *HMGCR* | A | G | 0.341 | 0.086 | 0.011 | 1.35E-15 | 63.9 | 20690 | 0.012 | 0.013 | 0.348 |
| rs35529421 | *ANGPTL3* | A | T | 0.299 | -0.062 | 0.011 | 1.67E-08 | 31.9 | 20684 | 0.011 | 0.017 | 0.510 |
| rs1367117 | *APOB* | A | G | 0.288 | 0.109 | 0.011 | 9.99E-22 | 91.9 | 20686 | -0.009 | 0.010 | 0.395 |
| rs964184 | *APOA5/APOC3* | C | G | 0.861 | -0.166 | 0.014 | 2.58E-30 | 131.3 | 20686 | -0.039 | 0.014 | 0.005 |
| rs115849089 | *LPL* | A | G | 0.107 | -0.100 | 0.017 | 1.04E-08 | 32.8 | 20686 | -0.007 | 0.015 | 0.662 |

*Abbreviations: EAF – effect allele frequency (frequencies in exposure GWAS are reported); Exposure – statistics refer to data extracted from GWAS of lipids; F – F statistic from the SNP-lipid association model; N – sample size from the SNP-lipid association model; Outcome -- statistics refer to data extracted from GWAS of PD risk*

Footnotes:

1. *F* statistics from SNP-exposure association testing are indicators of instrument strength in MR analyses – values under 10 may imply notable weak instrument bias could affect models. *F* statistics were estimated for SNP-lipid associations from the *F* distribution using the sample sizes and *P* values from the GWAS findings. In some instances, *F* values were recorded as ‘infinite’ where *P* values were extremely low (below 1.00×10^-200^). All values of *F* exceeded 32*,* indicating that these models were unlikely to suffer from weak instrument bias.

# Supplemental table 6: information on variants used to assess the effects of lowering circulating LDL-C, triglycerides and ApoB on PD with standard MR models

| **SNP** | **Effect allele** | **Other allele** | **EAF exposure** | **Beta exposure** | **SE exposure** | ***P* value exposure** | ***F***  **exposure** | **N exposure** | **Beta outcome** | **SE**  **outcome** | ***P* value outcome** |  |
| --- | --- | --- | --- | --- | --- | --- | --- | --- | --- | --- | --- | --- |
|  |  |  |  |  |  |  |  |  |  |  |  |  |
| *SNPS used to instrument variation in circulating LDL-C* | | | | | | | | | | | | |
| rs1016988 | C | T | 0.220 | -0.020 | 0.003 | 7.81E-10 | 37.8 | 295826 | 0.022 | 0.012 | 0.056 |  |
| rs10490626 | A | G | 0.069 | -0.053 | 0.005 | 1.29E-22 | 95.8 | 274383 | -0.016 | 0.018 | 0.376 |  |
| rs10885997 | G | A | 0.410 | 0.015 | 0.003 | 8.91E-08 | 28.6 | 258146 | 0.010 | 0.010 | 0.331 |  |
| rs11065987 | G | A | 0.364 | -0.026 | 0.003 | 8.01E-19 | 78.5 | 276356 | 0.002 | 0.010 | 0.819 |  |
| rs11136343 | G | A | 0.383 | 0.029 | 0.003 | 6.58E-26 | 110.8 | 289480 | 0.021 | 0.010 | 0.038 |  |
| rs11220462 | A | G | 0.145 | 0.043 | 0.005 | 3.64E-21 | 89.2 | 201852 | -0.008 | 0.014 | 0.555 |  |
| rs1169288 | C | A | 0.331 | 0.037 | 0.003 | 1.84E-35 | 154.5 | 271307 | 0.009 | 0.010 | 0.409 |  |
| rs1250229 | C | T | 0.741 | 0.015 | 0.003 | 5.46E-07 | 25.1 | 295826 | 0.002 | 0.011 | 0.853 |  |
| rs12748152 | T | C | 0.072 | 0.031 | 0.005 | 2.29E-09 | 35.7 | 292592 | 0.000 | 0.018 | 0.996 |  |
| rs1564348 | C | T | 0.153 | 0.047 | 0.004 | 2.11E-38 | 168.0 | 295826 | 0.028 | 0.013 | 0.036 |  |
| rs17404153 | T | G | 0.127 | -0.018 | 0.004 | 7.26E-06 | 20.1 | 283592 | 0.009 | 0.015 | 0.536 |  |
| rs174546 | T | C | 0.313 | -0.053 | 0.003 | 1.00E-200 | Infinite | 293792 | -0.013 | 0.010 | 0.202 |  |
| rs1800562 | A | G | 0.049 | -0.044 | 0.006 | 2.61E-12 | 49.0 | 291354 | 0.000 | 0.021 | 0.989 |  |
| rs1801689 | C | A | 0.027 | 0.105 | 0.008 | 1.97E-37 | 163.5 | 295826 | -0.005 | 0.030 | 0.857 |  |
| rs1891110 | A | G | 0.548 | 0.021 | 0.003 | 7.69E-15 | 60.4 | 295826 | -0.014 | 0.010 | 0.142 |  |
| rs2000999 | A | G | 0.209 | 0.063 | 0.004 | 1.00E-200 | Infinite | 262517 | -0.021 | 0.012 | 0.085 |  |
| rs2030746 | T | C | 0.413 | 0.014 | 0.003 | 1.88E-07 | 27.2 | 295826 | 0.008 | 0.010 | 0.412 |  |
| rs2076674 | C | T | 0.354 | 0.018 | 0.003 | 8.08E-11 | 42.2 | 285068 | 0.020 | 0.010 | 0.044 |  |
| rs2081687 | C | T | 0.663 | -0.028 | 0.003 | 3.76E-23 | 98.2 | 295826 | -0.014 | 0.011 | 0.175 |  |
| rs2125345 | C | T | 0.337 | -0.024 | 0.003 | 4.74E-16 | 65.9 | 276799 | 0.002 | 0.010 | 0.836 |  |
| rs2131925 | T | G | 0.648 | 0.044 | 0.003 | 1.00E-200 | Infinite | 295302 | 0.004 | 0.010 | 0.671 |  |
| rs2239619 | A | C | 0.619 | 0.018 | 0.003 | 7.42E-11 | 42.4 | 284338 | -0.005 | 0.011 | 0.643 |  |
| rs2255141 | G | A | 0.728 | -0.028 | 0.003 | 7.68E-21 | 87.7 | 295826 | 0.028 | 0.011 | 0.007 |  |
| rs2328223 | C | A | 0.249 | 0.030 | 0.005 | 5.63E-09 | 34.0 | 170762 | 0.002 | 0.013 | 0.877 |  |
| rs267733 | G | A | 0.137 | -0.025 | 0.004 | 5.59E-11 | 43.0 | 294565 | 0.037 | 0.013 | 0.005 |  |
| rs28929474 | T | C | 0.015 | 0.081 | 0.011 | 4.30E-14 | 57.0 | 290263 | -0.060 | 0.040 | 0.135 |  |
| rs314253 | C | T | 0.351 | -0.020 | 0.003 | 1.10E-12 | 50.7 | 295826 | 0.007 | 0.011 | 0.478 |  |
| rs3177928 | A | G | 0.138 | 0.035 | 0.004 | 6.10E-17 | 70.0 | 253199 | 0.066 | 0.014 | 0.000 |  |
| rs351855 | A | G | 0.289 | -0.018 | 0.003 | 3.94E-08 | 30.2 | 233058 | 0.007 | 0.011 | 0.495 |  |
| rs364585 | G | A | 0.639 | 0.019 | 0.003 | 3.99E-11 | 43.6 | 276356 | -0.008 | 0.010 | 0.421 |  |
| rs3764261 | A | C | 0.313 | -0.032 | 0.003 | 1.48E-29 | 127.5 | 289809 | -0.016 | 0.010 | 0.113 |  |
| rs3780181 | G | A | 0.075 | -0.037 | 0.005 | 1.80E-13 | 54.2 | 295826 | 0.013 | 0.019 | 0.498 |  |
| rs3812594 | A | G | 0.238 | -0.018 | 0.003 | 1.54E-08 | 32.0 | 293723 | 0.027 | 0.011 | 0.013 |  |
| rs4253772 | T | C | 0.096 | 0.018 | 0.005 | 0.0001014 | 15.1 | 276356 | -0.008 | 0.015 | 0.602 |  |
| rs4420638 | G | A | 0.178 | 0.168 | 0.004 | 1.00E-200 | Infinite | 199527 | -0.013 | 0.015 | 0.387 |  |
| rs4530754 | A | G | 0.556 | 0.017 | 0.003 | 9.50E-10 | 37.4 | 276356 | -0.014 | 0.010 | 0.134 |  |
| rs4722551 | C | T | 0.158 | 0.040 | 0.004 | 2.13E-26 | 113.0 | 275110 | 0.001 | 0.013 | 0.950 |  |
| rs4942486 | C | T | 0.516 | -0.022 | 0.003 | 4.48E-16 | 66.0 | 291053 | 0.008 | 0.010 | 0.382 |  |
| rs5763662 | T | C | 0.042 | 0.025 | 0.008 | 0.001783 | 9.8 | 265024 | 0.007 | 0.032 | 0.826 |  |
| rs61754230 | T | C | 0.016 | 0.057 | 0.011 | 1.14E-07 | 28.1 | 292762 | 0.228 | 0.062 | 0.000 |  |
| rs629301 | T | G | 0.774 | 0.158 | 0.003 | 1.00E-200 | Infinite | 295826 | -0.002 | 0.012 | 0.887 |  |
| rs6511720 | T | G | 0.109 | -0.211 | 0.004 | 1.00E-200 | Infinite | 295826 | -0.005 | 0.015 | 0.763 |  |
| rs6756629 | A | G | 0.063 | -0.111 | 0.005 | 1.00E-200 | Infinite | 295826 | -0.009 | 0.020 | 0.649 |  |
| rs6831256 | G | A | 0.442 | 0.013 | 0.003 | 2.28E-06 | 22.3 | 291053 | 0.009 | 0.010 | 0.358 |  |
| rs6882076 | C | T | 0.623 | 0.039 | 0.003 | 1.00E-200 | Infinite | 295826 | 0.004 | 0.010 | 0.709 |  |
| rs704 | A | G | 0.488 | 0.021 | 0.003 | 5.58E-16 | 65.6 | 295826 | -0.019 | 0.010 | 0.060 |  |
| rs7640978 | T | C | 0.094 | -0.033 | 0.005 | 6.15E-13 | 51.8 | 295826 | -0.031 | 0.017 | 0.074 |  |
| rs8017377 | A | G | 0.425 | 0.023 | 0.003 | 6.03E-17 | 70.0 | 295826 | -0.007 | 0.010 | 0.449 |  |
| rs9370867 | G | A | 0.530 | -0.033 | 0.003 | 3.02E-34 | 148.9 | 290263 | 0.009 | 0.010 | 0.325 |  |
| rs964184 | C | G | 0.847 | -0.037 | 0.004 | 1.13E-23 | 100.6 | 295826 | -0.039 | 0.014 | 0.005 |  |
| rs9646133 | T | G | 0.326 | -0.019 | 0.003 | 2.35E-11 | 44.7 | 295826 | -0.020 | 0.011 | 0.067 |  |
| rs9987289 | G | A | 0.899 | 0.057 | 0.005 | 4.28E-36 | 157.4 | 278685 | -0.009 | 0.021 | 0.664 |  |
| *SNPS used to instrument variation in circulating triglycerides* | | | | | | | | | | | | |
| rs1011731 | A | G | 0.559 | -0.015 | 0.003 | 2.37E-08 | 31.2 | 299453 | -0.012 | 0.010 | 0.247 |  |
| rs10440120 | C | A | 0.833 | 0.031 | 0.004 | 5.34E-11 | 43.1 | 174886 | -0.013 | 0.014 | 0.342 |  |
| rs10489615 | G | A | 0.567 | -0.039 | 0.003 | 4.21E-49 | 217.0 | 305168 | 0.000 | 0.010 | 0.965 |  |
| rs10861661 | C | A | 0.233 | 0.019 | 0.003 | 2.67E-09 | 35.4 | 281951 | -0.009 | 0.011 | 0.416 |  |
| rs10889353 | C | A | 0.330 | -0.077 | 0.003 | 6.39E-170 | Infinite | 304422 | -0.004 | 0.010 | 0.667 |  |
| rs11057401 | A | T | 0.302 | -0.028 | 0.003 | 7.18E-23 | 96.9 | 304964 | 0.013 | 0.010 | 0.198 |  |
| rs1106766 | T | C | 0.211 | -0.030 | 0.003 | 1.45E-20 | 86.4 | 305699 | -0.009 | 0.012 | 0.429 |  |
| rs11216382 | C | T | 0.162 | -0.021 | 0.004 | 2.47E-09 | 35.6 | 305699 | -0.005 | 0.013 | 0.699 |  |
| rs1126673 | T | C | 0.720 | 0.017 | 0.003 | 9.95E-09 | 32.9 | 305699 | -0.016 | 0.010 | 0.116 |  |
| rs1140085 | A | G | 0.127 | -0.023 | 0.004 | 8.65E-09 | 33.1 | 305699 | 0.012 | 0.015 | 0.406 |  |
| rs116843064 | A | G | 0.019 | -0.273 | 0.010 | 4.19E-175 | Infinite | 280371 | 0.046 | 0.050 | 0.356 |  |
| rs11871606 | A | C | 0.500 | 0.016 | 0.003 | 5.82E-10 | 38.4 | 305699 | 0.005 | 0.010 | 0.568 |  |
| rs12355784 | A | C | 0.473 | -0.030 | 0.003 | 4.96E-31 | 134.2 | 305699 | 0.005 | 0.010 | 0.589 |  |
| rs12545984 | C | T | 0.846 | 0.044 | 0.004 | 1.20E-23 | 100.5 | 177747 | -0.029 | 0.013 | 0.022 |  |
| rs12602912 | T | C | 0.214 | 0.023 | 0.003 | 1.61E-13 | 54.4 | 305699 | -0.044 | 0.012 | 0.000 |  |
| rs1260326 | C | T | 0.626 | -0.121 | 0.003 | 1.00E-200 | Infinite | 305699 | 0.018 | 0.010 | 0.065 |  |
| rs12748152 | T | C | 0.072 | 0.031 | 0.005 | 1.22E-09 | 36.9 | 302408 | 0.000 | 0.018 | 0.996 |  |
| rs12970134 | A | G | 0.256 | 0.017 | 0.003 | 1.10E-08 | 32.7 | 305699 | -0.014 | 0.011 | 0.192 |  |
| rs13107325 | T | C | 0.053 | 0.034 | 0.006 | 1.52E-08 | 32.0 | 303685 | -0.106 | 0.019 | 0.000 |  |
| rs13326165 | G | A | 0.804 | 0.020 | 0.003 | 9.69E-10 | 37.4 | 305699 | -0.008 | 0.012 | 0.509 |  |
| rs13389219 | T | C | 0.391 | -0.037 | 0.003 | 2.59E-39 | 172.1 | 279052 | -0.013 | 0.010 | 0.196 |  |
| rs1344642 | A | G | 0.437 | -0.015 | 0.003 | 1.42E-08 | 32.2 | 305699 | 0.001 | 0.010 | 0.907 |  |
| rs1495741 | A | G | 0.753 | -0.035 | 0.003 | 8.10E-31 | 133.2 | 305699 | 0.006 | 0.011 | 0.589 |  |
| rs1532085 | G | A | 0.593 | -0.031 | 0.003 | 4.61E-32 | 138.9 | 305699 | 0.013 | 0.010 | 0.208 |  |
| rs16826069 | G | A | 0.207 | 0.025 | 0.003 | 9.30E-15 | 60.0 | 304422 | 0.005 | 0.012 | 0.700 |  |
| rs174546 | T | C | 0.313 | 0.052 | 0.003 | 1.57E-74 | 333.8 | 303618 | -0.013 | 0.010 | 0.202 |  |
| rs1800588 | T | C | 0.245 | 0.047 | 0.003 | 8.58E-54 | 238.5 | 305699 | 0.004 | 0.012 | 0.751 |  |
| rs1801177 | A | G | 0.017 | 0.169 | 0.010 | 1.12E-61 | 274.7 | 304596 | -0.040 | 0.038 | 0.302 |  |
| rs180349 | A | T | 0.301 | 0.030 | 0.004 | 9.22E-14 | 55.5 | 161419 | 0.002 | 0.011 | 0.831 |  |
| rs1832007 | A | G | 0.868 | 0.033 | 0.005 | 1.72E-12 | 49.8 | 177504 | 0.013 | 0.014 | 0.341 |  |
| rs188247550 | T | C | 0.034 | -0.189 | 0.033 | 1.01E-08 | 32.9 | 19268 | 0.069 | 0.088 | 0.438 |  |
| rs1883025 | T | C | 0.263 | -0.022 | 0.003 | 1.20E-13 | 55.0 | 305699 | -0.019 | 0.011 | 0.082 |  |
| rs2068888 | A | G | 0.468 | -0.032 | 0.003 | 4.27E-34 | 148.2 | 305699 | -0.006 | 0.010 | 0.542 |  |
| rs2081687 | C | T | 0.663 | -0.019 | 0.003 | 1.25E-11 | 45.9 | 305699 | -0.014 | 0.011 | 0.175 |  |
| rs2167079 | T | C | 0.345 | -0.020 | 0.003 | 2.68E-12 | 48.9 | 305699 | -0.008 | 0.011 | 0.480 |  |
| rs2251824 | A | G | 0.167 | 0.023 | 0.004 | 1.06E-10 | 41.7 | 305699 | -0.037 | 0.014 | 0.007 |  |
| rs2287922 | A | G | 0.473 | 0.019 | 0.003 | 4.78E-12 | 47.8 | 276022 | 0.000 | 0.010 | 0.976 |  |
| rs2292642 | T | C | 0.607 | -0.020 | 0.003 | 3.44E-14 | 57.5 | 305699 | -0.015 | 0.010 | 0.128 |  |
| rs247616 | T | C | 0.308 | -0.036 | 0.003 | 2.42E-38 | 167.7 | 305699 | -0.017 | 0.010 | 0.097 |  |
| rs26008 | C | T | 0.921 | -0.028 | 0.005 | 5.34E-09 | 34.1 | 305699 | -0.027 | 0.020 | 0.172 |  |
| rs264 | A | G | 0.143 | -0.112 | 0.004 | 1.00E-200 | Infinite | 305699 | -0.009 | 0.014 | 0.496 |  |
| rs2745353 | T | C | 0.525 | 0.020 | 0.003 | 3.26E-15 | 62.1 | 305699 | 0.008 | 0.010 | 0.394 |  |
| rs2785990 | T | C | 0.685 | 0.016 | 0.003 | 1.24E-08 | 32.4 | 305699 | -0.002 | 0.010 | 0.878 |  |
| rs2792751 | C | T | 0.728 | 0.020 | 0.003 | 1.44E-11 | 45.6 | 305699 | 0.028 | 0.011 | 0.009 |  |
| rs2925979 | C | T | 0.698 | -0.029 | 0.003 | 1.24E-24 | 105.0 | 305699 | 0.020 | 0.011 | 0.071 |  |
| rs2943641 | C | T | 0.660 | 0.033 | 0.003 | 4.85E-33 | 143.4 | 305699 | 0.013 | 0.010 | 0.187 |  |
| rs3130564 | T | C | 0.163 | -0.033 | 0.004 | 5.44E-18 | 74.7 | 305699 | -0.008 | 0.013 | 0.534 |  |
| rs35332062 | A | G | 0.117 | -0.124 | 0.004 | 1.00E-200 | Infinite | 305699 | 0.038 | 0.019 | 0.041 |  |
| rs35665085 | A | G | 0.050 | 0.032 | 0.006 | 5.00E-08 | 29.7 | 302582 | -0.012 | 0.029 | 0.693 |  |
| rs3748034 | T | G | 0.135 | 0.035 | 0.004 | 4.65E-17 | 70.5 | 244109 | 0.017 | 0.014 | 0.216 |  |
| rs3760627 | C | T | 0.468 | 0.019 | 0.003 | 5.29E-09 | 34.1 | 176201 | 0.005 | 0.010 | 0.638 |  |
| rs3769823 | G | A | 0.690 | 0.017 | 0.003 | 1.36E-09 | 36.7 | 295956 | -0.006 | 0.010 | 0.578 |  |
| rs3803357 | A | C | 0.542 | -0.017 | 0.003 | 1.21E-10 | 41.4 | 305699 | 0.004 | 0.010 | 0.674 |  |
| rs38855 | A | G | 0.526 | 0.019 | 0.003 | 2.11E-08 | 31.4 | 177825 | -0.009 | 0.010 | 0.341 |  |
| rs4014195 | G | C | 0.346 | 0.016 | 0.003 | 7.86E-09 | 33.3 | 305699 | 0.024 | 0.010 | 0.014 |  |
| rs4149056 | C | T | 0.142 | 0.029 | 0.004 | 2.99E-14 | 57.7 | 296828 | 0.003 | 0.013 | 0.834 |  |
| rs4245791 | T | C | 0.718 | -0.019 | 0.003 | 3.37E-10 | 39.5 | 279029 | 0.015 | 0.010 | 0.160 |  |
| rs439401 | C | T | 0.626 | 0.075 | 0.003 | 2.68E-168 | Infinite | 305699 | -0.001 | 0.010 | 0.906 |  |
| rs4410790 | C | T | 0.593 | 0.015 | 0.003 | 1.17E-08 | 32.5 | 305699 | 0.023 | 0.010 | 0.020 |  |
| rs442177 | T | G | 0.575 | 0.031 | 0.003 | 4.02E-31 | 134.6 | 305699 | -0.006 | 0.010 | 0.541 |  |
| rs4722551 | C | T | 0.158 | -0.026 | 0.004 | 4.40E-12 | 47.9 | 283356 | 0.001 | 0.013 | 0.950 |  |
| rs4938303 | T | C | 0.677 | -0.102 | 0.003 | 1.00E-200 | Infinite | 284767 | -0.013 | 0.011 | 0.238 |  |
| rs4976033 | G | A | 0.426 | 0.018 | 0.003 | 1.71E-11 | 45.3 | 305699 | -0.010 | 0.010 | 0.324 |  |
| rs55707100 | T | C | 0.025 | 0.131 | 0.008 | 8.60E-54 | 238.5 | 303685 | -0.035 | 0.031 | 0.265 |  |
| rs58542926 | T | C | 0.073 | -0.118 | 0.005 | 3.73E-125 | Infinite | 305699 | 0.008 | 0.019 | 0.673 |  |
| rs6062343 | A | G | 0.444 | -0.018 | 0.003 | 4.07E-11 | 43.6 | 288510 | 0.018 | 0.010 | 0.072 |  |
| rs6066141 | T | C | 0.759 | 0.030 | 0.005 | 2.34E-08 | 31.2 | 89485 | 0.006 | 0.012 | 0.600 |  |
| rs61995676 | T | C | 0.020 | 0.093 | 0.009 | 2.70E-23 | 98.9 | 299984 | -0.039 | 0.048 | 0.417 |  |
| rs643381 | A | C | 0.496 | -0.023 | 0.003 | 1.96E-18 | 76.7 | 300795 | 0.010 | 0.012 | 0.419 |  |
| rs645040 | T | G | 0.781 | 0.023 | 0.003 | 6.23E-13 | 51.8 | 305699 | 0.016 | 0.014 | 0.271 |  |
| rs676210 | A | G | 0.253 | -0.071 | 0.003 | 4.94E-118 | Infinite | 305699 | -0.005 | 0.012 | 0.681 |  |
| rs6882076 | C | T | 0.623 | 0.038 | 0.003 | 1.23E-44 | 196.5 | 305699 | 0.004 | 0.010 | 0.709 |  |
| rs6995541 | G | A | 0.322 | 0.027 | 0.004 | 1.34E-12 | 50.3 | 177486 | -0.003 | 0.011 | 0.822 |  |
| rs7157785 | T | G | 0.177 | 0.023 | 0.004 | 6.37E-10 | 38.2 | 261253 | 0.002 | 0.013 | 0.865 |  |
| rs7200543 | G | A | 0.307 | 0.024 | 0.003 | 5.64E-17 | 70.1 | 284767 | -0.012 | 0.010 | 0.239 |  |
| rs7248104 | A | G | 0.404 | -0.020 | 0.003 | 1.81E-14 | 58.7 | 305699 | -0.014 | 0.010 | 0.138 |  |
| rs72836561 | T | C | 0.027 | 0.133 | 0.008 | 5.93E-64 | 285.2 | 302582 | 0.021 | 0.040 | 0.603 |  |
| rs738322 | G | A | 0.490 | -0.020 | 0.003 | 3.38E-14 | 57.5 | 305699 | 0.002 | 0.010 | 0.882 |  |
| rs749671 | G | A | 0.606 | 0.021 | 0.003 | 6.11E-10 | 38.3 | 176205 | -0.075 | 0.010 | 0.000 |  |
| rs7679 | C | T | 0.168 | 0.053 | 0.003 | 2.39E-53 | 236.5 | 305699 | 0.009 | 0.012 | 0.472 |  |
| rs7758229 | T | G | 0.305 | 0.018 | 0.003 | 4.54E-10 | 38.9 | 293886 | 0.004 | 0.010 | 0.673 |  |
| rs7940646 | C | T | 0.720 | 0.016 | 0.003 | 3.72E-08 | 30.3 | 305699 | 0.038 | 0.010 | 0.000 |  |
| rs7946 | T | C | 0.669 | -0.016 | 0.003 | 1.15E-08 | 32.6 | 304420 | 0.002 | 0.011 | 0.883 |  |
| rs8050136 | A | C | 0.392 | 0.017 | 0.003 | 8.98E-11 | 42.0 | 305699 | -0.007 | 0.010 | 0.503 |  |
| rs8182584 | G | T | 0.588 | -0.016 | 0.003 | 6.20E-09 | 33.8 | 268306 | 0.011 | 0.010 | 0.277 |  |
| rs9686661 | T | C | 0.189 | 0.042 | 0.003 | 1.99E-37 | 163.5 | 305699 | 0.007 | 0.012 | 0.572 |  |
| rs972283 | G | A | 0.555 | 0.027 | 0.003 | 6.40E-25 | 106.3 | 305699 | -0.009 | 0.010 | 0.385 |  |
| rs998584 | A | C | 0.482 | 0.034 | 0.003 | 1.22E-35 | 155.3 | 286938 | -0.005 | 0.010 | 0.610 |  |
| *SNPS used to instrument variation in circulating ApoB* | | | | | | | | | | | | |
| rs10056811 | A | G | 0.341 | 0.086 | 0.011 | 1.35E-15 | 63.9 | 20690 | 0.012 | 0.013 | 0.348 |  |
| rs1081105 | C | A | 0.020 | 0.223 | 0.039 | 2.32E-08 | 31.2 | 18400 | 0.019 | 0.032 | 0.552 |  |
| rs115849089 | A | G | 0.107 | -0.100 | 0.017 | 1.04E-08 | 32.8 | 20686 | -0.007 | 0.015 | 0.662 |  |
| rs11591147 | T | G | 0.031 | -0.438 | 0.035 | 2.50E-34 | 150.0 | 15270 | 0.036 | 0.043 | 0.405 |  |
| rs1260326 | C | T | 0.639 | -0.067 | 0.010 | 2.51E-10 | 40.1 | 20690 | 0.018 | 0.010 | 0.065 |  |
| rs1367117 | A | G | 0.288 | 0.109 | 0.011 | 9.99E-22 | 91.9 | 20686 | -0.009 | 0.010 | 0.395 |  |
| rs142130958 | A | G | 0.106 | -0.200 | 0.017 | 7.78E-32 | 138.3 | 20686 | -0.007 | 0.015 | 0.623 |  |
| rs144064722 | G | A | 0.025 | 0.199 | 0.035 | 2.29E-08 | 31.3 | 18398 | 0.024 | 0.041 | 0.561 |  |
| rs150617279 | A | T | 0.110 | -0.112 | 0.018 | 3.97E-10 | 39.2 | 18400 | -0.031 | 0.029 | 0.274 |  |
| rs182695896 | C | A | 0.019 | 0.237 | 0.042 | 2.49E-08 | 31.1 | 18398 | -0.047 | 0.094 | 0.622 |  |
| rs1883711 | C | G | 0.057 | 0.144 | 0.025 | 1.95E-08 | 31.6 | 18396 | -0.006 | 0.043 | 0.898 |  |
| rs190934192 | A | G | 0.024 | -0.320 | 0.041 | 1.30E-14 | 59.5 | 18398 | 0.002 | 0.071 | 0.974 |  |
| rs2495477 | G | A | 0.419 | -0.062 | 0.011 | 3.14E-08 | 30.6 | 20687 | -0.001 | 0.011 | 0.939 |  |
| rs2980875 | G | A | 0.484 | -0.070 | 0.010 | 6.68E-12 | 47.2 | 20688 | 0.016 | 0.010 | 0.096 |  |
| rs3005923 | A | G | 0.028 | -0.283 | 0.037 | 4.73E-14 | 56.9 | 18396 | 0.053 | 0.169 | 0.755 |  |
| rs629301 | T | G | 0.780 | 0.090 | 0.012 | 3.56E-13 | 52.9 | 20690 | -0.002 | 0.012 | 0.887 |  |
| rs635634 | T | C | 0.198 | 0.074 | 0.013 | 7.71E-09 | 33.4 | 20687 | -0.005 | 0.012 | 0.682 |  |
| rs6756629 | A | G | 0.078 | -0.113 | 0.019 | 1.90E-09 | 36.1 | 20690 | -0.009 | 0.020 | 0.649 |  |
| rs7412 | T | C | 0.056 | -0.428 | 0.026 | 4.39E-59 | 264.7 | 17554 | 0.000 | 0.018 | 0.987 |  |
| rs964184 | C | G | 0.861 | -0.166 | 0.014 | 2.58E-30 | 131.3 | 20686 | -0.039 | 0.014 | 0.005 |  |

*Abbreviations: EAF – effect allele frequency (frequencies in exposure GWAS are reported); Exposure – statistics refer to data extracted from GWAS of lipids; F – F statistic from the SNP-lipid association model; N – sample size from the SNP-lipid association model; Outcome -- statistics refer to data extracted from GWAS of PD risk*

Footnotes:

1. *F* statistics from SNP-exposure association testing are indicators of instrument strength in MR analyses – values under 10 may imply notable weak instrument bias could affect models. *F* statistics were estimated for SNP-lipid associations from the *F* distribution using the sample sizes and *P* values from the GWAS findings.
2. In some instances, *F* values were recorded as ‘infinite’ where *P* values were extremely low (below 1.00×10^-200^). All values of *F* were equal to or higher than 10*,* indicating that these models were unlikely to suffer from weak instrument bias.
3. Some *P* values for SNP-LDL associations are not below genome-wide significance threshold (5e-08): these are either discovery sample *P* values that were replicated in additional data in the GLGC exome-wide study, or exome-wide results that had been reported as having *P* values under 5e-08 in the previous GLGC GWAS (in which case, exome-wide estimates were prioritised due to these being derived from larger sample sizes)

# **Supplemental table 7** – MR estimates for the effects of lowering LDL-C, triglycerides and ApoB on PD risk

| **Exposure** | **# SNPs** | **Method** | **Odds ratio** | **95% CI** | ***P*** |
| --- | --- | --- | --- | --- | --- |
| LDL | 52 | Inverse variance weighted | 0.99 | (0.89, 1.09) |  |
|  |  | MR Egger | 0.98 | (0.85, 1.14) |  |
|  |  | *MR Egger intercept test* |  |  | 0.91 |
|  |  | Weighted median | 1.00 | (0.91, 1.10) |  |
|  |  |  |  |  |  |
| Triglycerides | 88 | Inverse variance weighted | 1.06 | (0.96, 1.16) |  |
|  |  | MR Egger | 1.07 | (0.91, 1.25) |  |
|  |  | *MR Egger intercept test* |  |  | 0.85 |
|  |  | Weighted median | 1.02 | (0.92, 1.12) |  |
|  |  |  |  |  |  |
| ApoB | 20 | Inverse variance weighted | 0.99 | (0.94, 1.04) |  |
|  |  | MR Egger | 0.95 | (0.87, 1.04) |  |
|  |  | *MR Egger intercept test* |  |  | 0.30 |
|  |  | Weighted median | 1.00 | (0.93, 1.07) |  |

Footnotes:

1. All results are coded per standard deviation lower concentrations of the circulating biomarker – odds ratios above one indicating higher risk of PD with lower exposure to the lipid fraction. A low *P* value from the intercept test from the MR Egger method indicates evidence for overall (non-neutral/directional) bias from genetic pleiotropy, which could either inflate or attenuate the MR estimates.
2. I^2^_g-x_ metrics for the three sets of instruments were ≥ 96.8%

# Supplemental table 8: group authorship members

| **Author** | **Affiliation** |  |  |
| --- | --- | --- | --- |
|  |  |  |  |
| **The 23andMe Research Team** | |  |  |
| Michelle Agee, Adam Auton, Robert K. Bell, Katarzyna Bryc, Paul Cannon, Sarah L. Elson, Pierre Fontanillas, Nicholas A. Furlotte, Karl Heilbron, Barry Hicks, David A. Hinds, Karen E. Huber, Ethan M. Jewett, Yunxuan Jiang, Aaron Kleinman, Keng-Han Lin, Nadia K. Litterman, Jennifer C. McCreight, Matthew H. McIntyre, Kimberly F. McManus, Joanna L. Mountain, Elizabeth S. Noblin, Carrie A.M. Northover, Steven J. Pitts, G. David Poznik, J. Fah Sathirapongsasuti, Janie F. Shelton, Suyash Shringarpure, Chao Tian, Joyce Y. Tung, Vladimir Vacic, and Xin Wang. | 23andMe, Inc., Sunnyvale, California 94086, USA |  |  |
|  |  |  |  |
|  |  |  |  |
|  |  |  |  |
| **Members of the IPDGC (grouped by country of affiliation) *** | |  |  |
|  |  |  |  |
| *United Kingdom:* |  |  |  |
| Alastair J Noyce | Preventive Neurology Unit, Wolfson Institute of Preventive Medicine, QMUL, London, UK and Department of Molecular Neuroscience, UCL, London, UK |  |  |
| Rauan Kaiyrzhanov | Department of Molecular Neuroscience, UCL Institute of Neurology, London, UK |  |  |
| Ben Middlehurst | Institute of Translational Medicine, University of Liverpool, Liverpool, UK |  |  |
| Demis A Kia | UCL Genetics Institute; and Department of Molecular Neuroscience, UCL Institute of Neurology, London, UK |  |  |
| Manuela Tan | Department of Clinical Neuroscience, University College London, London, UK |  |  |
| Henry Houlden | Department of Molecular Neuroscience, UCL Institute of Neurology, London, UK |  |  |
| Catherine Storm | Department of Clinical and Movement Neurosciences, UCL Queen Square Institute of Neurology, London, UK |  |  |
| Huw R Morris | Department of Clinical Neuroscience, University College London, London, UK |  |  |
| Helene Plun-Favreau | Department of Molecular Neuroscience, UCL Institute of Neurology, London, UK |  |  |
| Peter Holmans | Biostatistics & Bioinformatics Unit, Institute of Psychological Medicine and Clinical Neuroscience, MRC Centre for Neuropsychiatric Genetics & Genomics, Cardiff, UK |  |  |
| John Hardy | Department of Molecular Neuroscience, UCL Institute of Neurology, London, UK |  |  |
| Daniah Trabzuni | Department of Molecular Neuroscience, UCL Institute of Neurology, London, UK; Department of Genetics, King Faisal Specialist Hospital and Research Centre, Riyadh, 11211 Saudi Arabia |  |  |
| John Quinn | Institute of Translational Medicine, University of Liverpool, Liverpool, UK |  |  |
| Vivien Bubb | Institute of Translational Medicine, University of Liverpool, Liverpool, UK |  |  |
| Kin Y Mok | Department of Molecular Neuroscience, UCL Institute of Neurology, London, UK |  |  |
| Kerri J. Kinghorn | Institute of Healthy Ageing, Research Department of Genetics, Evolution and Environment, University College London, London, UK |  |  |
| Nicholas W Wood | UCL Genetics Institute; and Department of Molecular Neuroscience, UCL Institute of Neurology, London, UK |  |  |
| Patrick Lewis | University of Reading, Reading, UK |  |  |
| Sebastian R Schreglmann | Department of Molecular Neuroscience, UCL Institute of Neurology, London, UK |  |  |
| Ruth Lovering | University College London, London, UK |  |  |
| Lea R’Bibo | Department of Molecular Neuroscience, UCL Institute of Neurology, London, UK |  |  |
| Claudia Manzoni | University of Reading, Reading, UK |  |  |
| Mie Rizig | Department of Molecular Neuroscience, UCL Institute of Neurology, London, UK |  |  |
| Mina Ryten | Department of Molecular Neuroscience, UCL Institute of Neurology, London, UK |  |  |
| Sebastian Guelfi | Department of Molecular Neuroscience, UCL Institute of Neurology, London, UK |  |  |
| Valentina Escott-Price | MRC Centre for Neuropsychiatric Genetics and Genomics, Cardiff University School of Medicine, Cardiff, UK |  |  |
| Viorica Chelban | Department of Molecular Neuroscience, UCL Institute of Neurology, London, UK |  |  |
| Thomas Foltynie | UCL Institute of Neurology, London, UK |  |  |
| Nigel Williams | MRC Centre for Neuropsychiatric Genetics and Genomics, Cardiff, UK |  |  |
| Karen E. Morrison | Faculty of Medicine, University of Southampton, UK |  |  |
| Carl Clarke | University of Birmingham, Birmingham, UK and Sandwell and West Birmingham Hospitals NHS Trust, Birmingham, UK |  |  |
| Kirsten Harvey | UCL School of Pharmacy, UK |  |  |
| Benjamin M Jacobs | Preventive Neurology Unit, Wolfson Institute of Preventive Medicine, QMUL, London, UK |  |  |
|  |  |  |  |
| *France:* |  |  |  |
| Alexis Brice | Institut du Cerveau et de la Moelle épinière, ICM, Inserm U 1127, CNRS, UMR 7225, Sorbonne Universités, UPMC University Paris 06, UMR S 1127, AP-HP, Pitié-Salpêtrière Hospital, Paris, France |  |  |
| Fabrice Danjou | Institut du Cerveau et de la Moelle épinière, ICM, Inserm U 1127, CNRS, UMR 7225, Sorbonne Universités, UPMC University Paris 06, UMR S 1127, AP-HP, Pitié-Salpêtrière Hospital, Paris, France |  |  |
| Suzanne Lesage | Institut du Cerveau et de la Moelle épinière, ICM, Inserm U 1127, CNRS, UMR 7225, Sorbonne Universités, UPMC University Paris 06, UMR S 1127, AP-HP, Pitié-Salpêtrière Hospital, Paris, France |  |  |
| Jean-Christophe Corvol | Institut du Cerveau et de la Moelle épinière, ICM, Inserm U 1127, CNRS, UMR 7225, Sorbonne Universités, UPMC University Paris 06, UMR S 1127; Centre d’Investigation Clinique Pitié Neurosciences CIC-1422, AP-HP, Pitié-Salpêtrière Hospital, Paris, France |  |  |
| Maria Martinez | INSERM UMR 1220; and Paul Sabatier University, Toulouse, France |  |  |
|  |  |  |  |
| *Germany:* |  |  |  |
| Claudia Schulte | Department for Neurodegenerative Diseases, Hertie Institute for Clinical Brain Research, University of Tübingen, and DZNE, German Center for Neurodegenerative Diseases, Tübingen, Germany |  |  |
| Kathrin Brockmann | Department for Neurodegenerative Diseases, Hertie Institute for Clinical Brain Research, University of Tübingen, and DZNE, German Center for Neurodegenerative Diseases, Tübingen, Germany |  |  |
| Javier Simón-Sánchez | Department for Neurodegenerative Diseases, Hertie Institute for Clinical Brain Research, University of Tübingen, and DZNE, German Center for Neurodegenerative Diseases, Tübingen, Germany |  |  |
| Peter Heutink | DZNE, German Center for Neurodegenerative Diseases and Department for Neurodegenerative Diseases, Hertie Institute for Clinical Brain Research, University of Tübingen, Tübingen, Germany |  |  |
| Patrizia Rizzu | DZNE, German Center for Neurodegenerative Diseases |  |  |
| Manu Sharma | Centre for Genetic Epidemiology, Institute for Clinical Epidemiology and Applied Biometry, University of Tubingen, Germany |  |  |
| Thomas Gasser | Department for Neurodegenerative Diseases, Hertie Institute for Clinical Brain Research, and DZNE, German Center for Neurodegenerative Diseases, Tübingen, Germany |  |  |
| Susanne A. Schneider | Department of Neurology, Ludwig-Maximilians-University Munich, München, Germany) |  |  |
|  |  |  |  |
| *United States of America:* |  |  |  |
| Mark R Cookson | Laboratory of Neurogenetics, National Institute on Aging, Bethesda, USA |  |  |
| Sara Bandres-Ciga | Laboratory of Neurogenetics, National Institute on Aging, Bethesda, MD, USA |  |  |
| Cornelis Blauwendraat | Laboratory of Neurogenetics, National Institute on Aging, Bethesda, MD, USA |  |  |
| David W. Craig | Department of Translational Genomics, Keck School of Medicine, University of Southern California, Los Angeles, USA |  |  |
| Kimberley Billingsley | Laboratory of Neurogenetics, National Institute on Aging, Bethesda, MD, USA |  |  |
| Mary B. Makarious | Laboratory of Neurogenetics, National Institute on Aging, Bethesda, MD, USA |  |  |
| Derek Narendra | Inherited Movement Disorders Unit, National pInstitute of Neurological Disorders and Stroke, Bethesda, MD, USA |  |  |
| Faraz Faghri | Laboratory of Neurogenetics, National Institute on Aging, Bethesda, USA; Department of Computer Science, University of Illinois at Urbana-Champaign, Urbana, IL, USA |  |  |
| J Raphael Gibbs | Laboratory of Neurogenetics, National Institute on Aging, National Institutes of Health, Bethesda, MD, USA |  |  |
| Dena G. Hernandez | Laboratory of Neurogenetics, National Institute on Aging, Bethesda, MD, USA |  |  |
| Kendall Van Keuren-Jensen | Neurogenomics Division, TGen, Phoenix, AZ USA |  |  |
| Joshua M. Shulman | Departments of Neurology, Neuroscience, and Molecular & Human Genetics, Baylor College of Medicine, Houston, Texas, USA; Jan and Dan Duncan Neurological Research Institute, Texas Children’s Hospital, Houston, Texas, USA |  |  |
| Hirotaka Iwaki | Laboratory of Neurogenetics, National Institute on Aging, Bethesda, MD, USA |  |  |
| Hampton L. Leonard | Laboratory of Neurogenetics, National Institute on Aging, Bethesda, MD, USA |  |  |
| Mike A. Nalls | Laboratory of Neurogenetics, National Institute on Aging, Bethesda, USA; CEO/Consultant Data Tecnica International, Glen Echo, MD, USA |  |  |
| Laurie Robak | Baylor College of Medicine, Houston, Texas, USA |  |  |
| Jose Bras | Center for Neurodegenerative Science, Van Andel Research Institute, Grand Rapids, Michigan, USA |  |  |
| Rita Guerreiro | Center for Neurodegenerative Science, Van Andel Research Institute, Grand Rapids, Michigan, USA | |  |
| Steven Lubbe | Ken and Ruth Davee Department of Neurology, Northwestern University Feinberg School of Medicine, Chicago, IL, USA |  |  |
| Steven Finkbeiner | Departments of Neurology and Physiology, University of California, San Francisco; Gladstone Institute of Neurological Disease; Taube/Koret Center for Neurodegenerative Disease Research, San Francisco, CA, USA |  |  |
| Niccolo E. Mencacci | Northwestern University Feinberg School of Medicine, Chicago, IL, USA |  |  |
| Codrin Lungu | National Institutes of Health Division of Clinical Research, NINDS, National Institutes of Health, Bethesda, MD, USA |  |  |
| Andrew B Singleton | Laboratory of Neurogenetics, National Institute on Aging, Bethesda, MD, USA |  |  |
| Sonja W. Scholz | Neurodegenerative Diseases Research Unit, National Institute of Neurological Disorders and Stroke, Bethesda, MD, USA |  |  |
| Xylena Reed | Laboratory of Neurogenetics, National Institute on Aging, Bethesda, MD, USA). |  |  |
| Roy N. Alcalay | Department of Neurology, College of Physicians and Surgeons, Columbia University Medical Center, New York, NY, USA, Taub Institute for Research on Alzheimer's Disease and the Aging Brain, College of Physicians and Surgeons, Columbia University Medical Center, New York, NY, USA. |  |  |
| Zbigniew K. Wszolek | Department of Neurology, Mayo Clinic Jacksonville, FL, USA |  |  |
| Ryan J. Uitti | Department of Neurology, Mayo Clinic Jacksonville, FL, USA |  |  |
| Owen A. Ross | Departments of Neuroscience & Clinical Genomics, Mayo Clinic Jacksonville, FL, USA |  |  |
|  |  |  |  |
| *Canada:* |  |  |  |
| Ziv Gan-Or | Montreal Neurological Institute and Hospital, Department of Neurology & Neurosurgery, Department of Human Genetics, McGill University, Montréal, QC, H3A 0G4, Canada |  |  |
| Guy A. Rouleau | Montreal Neurological Institute and Hospital, Department of Neurology & Neurosurgery, Department of Human Genetics, McGill University, Montréal, QC, H3A 0G4, Canada |  |  |
| Lynne Krohn | Montreal Neurological Institute and Hospital, Department of Neurology & Neurosurgery, Department of Human Genetics, McGill University, Montréal, QC, H3A 0G4, Canada |  |  |
| Kheireddin Mufti | Montreal Neurological Institute and Hospital, Department of Neurology & Neurosurgery, Department of Human Genetics, McGill University, Montréal, QC, H3A 0G4, Canada |  |  |
|  |  |  |  |
| *The Netherlands:* |  |  |  |
| Jacobus J van Hilten | Department of Neurology, Leiden University Medical Center, Leiden, Netherlands |  |  |
| Johan Marinus | Department of Neurology, Leiden University Medical Center, Leiden, Netherlands |  |  |
|  |  |  |  |
| *Spain:* |  |  |  |
| Astrid D. Adarmes-Gómez | Instituto de Biomedicina de Sevilla [IBiS], Hospital Universitario Virgen del Rocío/CSIC/Universidad de Sevilla, Seville |  |  |
| Miquel Aguilar | Fundació Docència i Recerca Mútua de Terrassa and Movement Disorders Unit, Department of Neurology, University Hospital Mutua de Terrassa, Terrassa, Barcelona. |  |  |
| Ignacio Alvarez | Fundació Docència i Recerca Mútua de Terrassa and Movement Disorders Unit, Department of Neurology, University Hospital Mutua de Terrassa, Terrassa, Barcelona. |  |  |
| Victoria Alvarez | Hospital Universitario Central de Asturias, Oviedo |  |  |
| Francisco Javier Barrero | Hospital Universitario San Cecilio de Granada, Universidad de Granada |  |  |
| Jesús Alberto Bergareche Yarza | Instituto de Investigación Sanitaria Biodonostia, San Sebastián |  |  |
| Inmaculada Bernal-Bernal | Instituto de Biomedicina de Sevilla [IBiS], Hospital Universitario Virgen del Rocío/CSIC/Universidad de Sevilla, Seville |  |  |
| Marta Blazquez | Hospital Universitario Central de Asturias, Oviedo |  |  |
| Marta Bonilla-Toribio | Instituto de Biomedicina de Sevilla [IBiS], Hospital Universitario Virgen del Rocío/CSIC/Universidad de Sevilla, Seville |  |  |
| Juan A. Botía | Universidad de Murcia, Murcia |  |  |
| María Teresa Boungiorno | Fundació Docència i Recerca Mútua de Terrassa and Movement Disorders Unit, Department of Neurology, University Hospital Mutua de Terrassa, Terrassa, Barcelona. |  |  |
| Dolores Buiza-Rueda | Instituto de Biomedicina de Sevilla [IBiS], Hospital Universitario Virgen del Rocío/CSIC/Universidad de Sevilla, Seville |  |  |
| Ana Cámara | Hospital Clinic de Barcelona |  |  |
| Fátima Carrillo | Instituto de Biomedicina de Sevilla [IBiS], Hospital Universitario Virgen del Rocío/CSIC/Universidad de Sevilla, Seville |  |  |
| Mario Carrión-Claro | Instituto de Biomedicina de Sevilla [IBiS], Hospital Universitario Virgen del Rocío/CSIC/Universidad de Sevilla, Seville |  |  |
| Debora Cerdan | Hospital General de Segovia, Segovia |  |  |
| Jordi Clarimón | Memory Unit, Department of Neurology, IIB Sant Pau, Hospital de la Santa Creu i Sant Pau, Universitat Autònoma de Barcelona and Centro de Investigación Biomédica en Red en Enfermedades Neurodegenerativas [CIBERNED], Madrid |  |  |
| Yaroslau Compta | Hospital Clinic de Barcelona |  |  |
| Monica Diez-Fairen | Fundació Docència i Recerca Mútua de Terrassa and Movement Disorders Unit, Department of Neurology, University Hospital Mutua de Terrassa, Terrassa, Barcelona. |  |  |
| Oriol Dols-Icardo | Memory Unit, Department of Neurology, IIB Sant Pau, Hospital de la Santa Creu i Sant Pau, Universitat Autònoma de Barcelona, Barcelona, and Centro de Investigación Biomédica en Red en Enfermedades Neurodegenerativas [CIBERNED], Madrid |  |  |
| Jacinto Duarte | Hospital General de Segovia, Segovia |  |  |
| Raquel Duran | Centro de Investigacion Biomedica, Universidad de Granada, Granada |  |  |
| Francisco Escamilla-Sevilla | Hospital Universitario Virgen de las Nieves, Instituto de Investigación Biosanitaria de Granada, Granada |  |  |
| Mario Ezquerra | Hospital Clinic de Barcelona |  |  |
| Cici Feliz | Departmento de Neurologia, Instituto de Investigación Sanitaria Fundación Jiménez Díaz, Madrid, Spain |  |  |
| Manel Fernández | Hospital Clinic de Barcelona |  |  |
| Rubén Fernández-Santiago | Hospital Clinic de Barcelona |  |  |
| Ciara Garcia | Hospital Universitario Central de Asturias, Oviedo |  |  |
| Pedro García-Ruiz | Instituto de Investigación Sanitaria Fundación Jiménez Díaz, Madrid |  |  |
| Pilar Gómez-Garre | Instituto de Biomedicina de Sevilla [IBiS], Hospital Universitario Virgen del Rocío/CSIC/Universidad de Sevilla, Seville |  |  |
| Maria Jose Gomez Heredia | Hospital Universitario Virgen de la Victoria, Malaga |  |  |
| Isabel Gonzalez-Aramburu | Hospital Universitario Marqués de Valdecilla-IDIVAL, Santander |  |  |
| Ana Gorostidi Pagola | Instituto de Investigación Sanitaria Biodonostia, San Sebastián |  |  |
| Janet Hoenicka | Institut de Recerca Sant Joan de Déu, Barcelona |  |  |
| Jon Infante | Hospital Universitario Marqués de Valdecilla-IDIVAL and University of Cantabria, Santander, and Centro de Investigación Biomédica en Red en Enfermedades Neurodegenerativas [CIBERNED] |  |  |
| Silvia Jesús | Instituto de Biomedicina de Sevilla [IBiS], Hospital Universitario Virgen del Rocío/CSIC/Universidad de Sevilla, Seville |  |  |
| Adriano Jimenez-Escrig | Hospital Universitario Ramón y Cajal, Madrid |  |  |
| Jaime Kulisevsky | Movement Disorders Unit, Department of Neurology, IIB Sant Pau, Hospital de la Santa Creu i Sant Pau, Universitat Autònoma de Barcelona, Barcelona, and Centro de Investigación Biomédica en Red en Enfermedades Neurodegenerativas [CIBERNED] |  |  |
| Miguel A. Labrador-Espinosa | Instituto de Biomedicina de Sevilla [IBiS], Hospital Universitario Virgen del Rocío/CSIC/Universidad de Sevilla, Seville |  |  |
| Jose Luis Lopez-Sendon | Hospital Universitario Ramón y Cajal, Madrid |  |  |
| Adolfo López de Munain Arregui | Instituto de Investigación Sanitaria Biodonostia, San Sebastián |  |  |
| Daniel Macias | Instituto de Biomedicina de Sevilla [IBiS], Hospital Universitario Virgen del Rocío/CSIC/Universidad de Sevilla, Seville |  |  |
| Irene Martínez Torres | Department of Neurology, Instituto de Investigación Sanitaria La Fe, Hospital Universitario y Politécnico La Fe, Valencia |  |  |
| Juan Marín | Movement Disorders Unit, Department of Neurology, IIB Sant Pau, Hospital de la Santa Creu i Sant Pau, Universitat Autònoma de Barcelona, Barcelona, and Centro de Investigación Biomédica en Red en Enfermedades Neurodegenerativas [CIBERNED] |  |  |
| Maria Jose Marti | Hospital Clinic Barcelona |  |  |
| Juan Carlos Martínez-Castrillo | Instituto Ramón y Cajal de Investigación Sanitaria, Hospital Universitario Ramón y Cajal, Madrid |  |  |
| Carlota Méndez-del-Barrio | Instituto de Biomedicina de Sevilla [IBiS], Hospital Universitario Virgen del Rocío/CSIC/Universidad de Sevilla, Seville |  |  |
| Manuel Menéndez González | Hospital Universitario Central de Asturias, Oviedo |  |  |
| Marina Mata | Department of Neurology, Hospital Universitario Infanta Sofía, Madrid, Spain |  |  |
| Adolfo Mínguez | Hospital Universitario Virgen de las Nieves, Granada, Instituto de Investigación Biosanitaria de Granada |  |  |
| Pablo Mir | Instituto de Biomedicina de Sevilla [IBiS], Hospital Universitario Virgen del Rocío/CSIC/Universidad de Sevilla, Seville |  |  |
| Elisabet Mondragon Rezola | Instituto de Investigación Sanitaria Biodonostia, San Sebastián |  |  |
| Esteban Muñoz | Hospital Clinic Barcelona |  |  |
| Javier Pagonabarraga | Movement Disorders Unit, Department of Neurology, IIB Sant Pau, Hospital de la Santa Creu i Sant Pau, Universitat Autònoma de Barcelona, Barcelona, and Centro de Investigación Biomédica en Red en Enfermedades Neurodegenerativas [CIBERNED] |  |  |
| Pau Pastor | Fundació Docència i Recerca Mútua de Terrassa and Movement Disorders Unit, Department of Neurology, University Hospital Mutua de Terrassa, Terrassa, Barcelona. |  |  |
| Francisco Perez Errazquin | Hospital Universitario Virgen de la Victoria, Malaga |  |  |
| Teresa Periñán-Tocino | Instituto de Biomedicina de Sevilla [IBiS], Hospital Universitario Virgen del Rocío/CSIC/Universidad de Sevilla, Seville |  |  |
| Javier Ruiz-Martínez | Hospital Universitario Donostia, Instituto de Investigación Sanitaria Biodonostia, San Sebastián |  |  |
| Clara Ruz | Centro de Investigacion Biomedica, Universidad de Granada, Granada |  |  |
| Antonio Sanchez Rodriguez | Hospital Universitario Marqués de Valdecilla-IDIVAL, Santander |  |  |
| María Sierra | Hospital Universitario Marqués de Valdecilla-IDIVAL, Santander |  |  |
| Esther Suarez-Sanmartin | Hospital Universitario Central de Asturias, Oviedo |  |  |
| Cesar Tabernero | Hospital General de Segovia, Segovia |  |  |
| Juan Pablo Tartari | Fundació Docència i Recerca Mútua de Terrassa and Movement Disorders Unit, Department of Neurology, University Hospital Mutua de Terrassa, Terrassa, Barcelona |  |  |
| Cristina Tejera-Parrado | Instituto de Biomedicina de Sevilla [IBiS], Hospital Universitario Virgen del Rocío/CSIC/Universidad de Sevilla, Seville |  |  |
| Eduard Tolosa | Hospital Clinic Barcelona |  |  |
| Francesc Valldeoriola | Hospital Clinic Barcelona |  |  |
| Laura Vargas-González | Instituto de Biomedicina de Sevilla [IBiS], Hospital Universitario Virgen del Rocío/CSIC/Universidad de Sevilla, Seville |  |  |
| Lydia Vela | Department of Neurology, Hospital Universitario Fundación Alcorcón, Madrid |  |  |
| Francisco Vives | Centro de Investigacion Biomedica, Universidad de Granada, Granada. |  |  |
|  |  |  |  |
| *Austria:* |  |  |  |
| Alexander Zimprich | Department of Neurology, Medical University of Vienna, Austria |  |  |
|  |  |  |  |
| *Norway:* |  |  |  |
| Lasse Pihlstrom | Department of Neurology, Oslo University Hospital, Oslo, Norway |  |  |
| Mathias Toft | Department of Neurology and Institute of Clinical Medicine, Oslo University Hospital, Oslo, Norway |  |  |
|  |  |  |  |
| *Estonia:* |  |  |  |
| Pille Taba | Department of Neurology and Neurosurgery, University of Tartu, Tartu, Estonia |  |  |
|  |  |  |  |
| *Australia:* |  |  |  |
| Sulev Koks | Centre for Molecular Medicine and Innovative Therapeutics, Murdoch University, Murdoch, 6150, Perth, Western Australia; The Perron Institute for Neurological and Translational Science, Nedlands, 6009, Perth, Western Australia |  |  |
|  |  |  |  |
| *Israel:* |  |  |  |
| Sharon Hassin-Baer | The Movement Disorders Institute, Department of Neurology and Sagol Neuroscience Center, Chaim Sheba Medical Center, Tel-Hashomer, 5262101, Ramat Gan, Israel, Sackler Faculty of Medicine, Tel Aviv University, Tel Aviv, Israel |  |  |
|  |  |  |  |
| *Finland:* |  |  |  |
| Kari Majamaa | Institute of Clinical Medicine, Department of Neurology, University of Oulu, Oulu, Finland; Department of Neurology and Medical Research Center, Oulu University Hospital, Oulu, Finland |  |  |
| Ari Siitonen | Institute of Clinical Medicine, Department of Neurology, University of Oulu, Oulu, Finland; Department of Neurology and Medical Research Center, Oulu University Hospital, Oulu, Finland |  |  |
| Pentti Tienari | Clinical Neurosciences, Neurology, University of Helsinki, Helsinki, Finland, Helsinki University Hospital, Helsinki, Finland |  |  |
|  |  |  |  |
| *Nigeria:* |  |  |  |
| Njideka U. Okubadejo | University of Lagos, Lagos State, Nigeria |  |  |
| Oluwadamilola O. Ojo | University of Lagos, Lagos State, Nigeria |  |  |
|  |  |  |  |
| *Kazakhstan:* |  |  |  |
| Coordinator - Rauan Kaiyrzhanov | Department of Molecular Neuroscience, UCL Institute of Neurology, London, UK |  |  |
| Chingiz Shashkin | Kazakh National Medical University named after Asfendiyarov, Almaty, Kazakhstan |  |  |
| Nazira Zharkinbekova | South Kazakhstan Medical Academy, Shymkent, Kazakhstan |  |  |
| Vadim Akhmetzhanov | Astana Medical University, Astana Kazakhstan |  |  |
| Akbota Aitkulova | National Center for Biotechnology, Astana, Kazakhstan; Al-Farabi Kazakh national university. Almaty Kazakhstan |  |  |
| Elena Zholdybayeva | National Center for Biotechnology, Astana, Kazakhstan |  |  |
| Zharkyn Zharmukhanov | National Center for Biotechnology, Astana, Kazakhstan |  |  |
| Gulnaz Kaishybayeva | Scientific and practical center “Institute of neurology named after Smagul Kaishibayev”, Almaty, Kazakhstan |  |  |
| Altynay Karimova | Scientific and practical center “Institute of neurology named after Smagul Kaishibayev”, Almaty, Kazakhstan |  |  |
| Talgat Khaibullin | Semey Medical University, Semey, Kazakhstan. |  |  |
|  |  |  |  |
| *Ireland:* |  |  |  |
| Timothy L. Lynch | The Dublin Neurological Institute at the Mater Misericordiae University Hospital, Dublin, Ireland & School of Medicine and Medical Science, University College Dublin, Dublin, Ireland |  |  |

* For a complete overview of IPDGC members, acknowledgements and funding, please visit http://pdgenetics.org/partners
